# Synergism of Specific Maca Phenotypes (*Lepidium peruvianum*) in Combination with Saw Palmetto (*Serenoa repens*) Extract for Chemoprevention of Prostate Cancer as Determined in In Vitro Cytotoxicity Assays on Human Epithelial and Prostate Cancer Cells

**DOI:** 10.3390/molecules29235632

**Published:** 2024-11-28

**Authors:** Katarzyna Gaweł-Bęben, Wirginia Kukula-Koch, Dominik Szwajgier, Beata Antosiewicz-Klimczak, Rita Cristina Orihuela-Campos, Kazimierz Głowniak, Henry O. Meissner

**Affiliations:** 1 Department of Cosmetology, University of Information Technology and Management in Rzeszow, Sucharskiego 2, 35-225 Rzeszow, Poland; bantosiewicz@wsiz.edu.pl; 2 Department of Pharmacognosy with Garden of Medicinal Plants, Medical University of Lublin, Chodźki 1, 20-093 Lublin, Poland; virginia.kukula@gmail.com; 3 Department of Biotechnology, Microbiology and Human Nutrition, University of Life Sciences, Skromna 8, 20-704 Lublin, Poland; dominik.szwajgier@up.lublin.pl; 4 Academic Department of Stomatology for Children and Adolescents, Integrated Faculties of Medicine, Stomatology and Nursing, Cayetano Heredia Peruvian University, Av. Honorio Delgado 430, Lima 15102, Peru; rita.orihuela.c@upch.pe; 5 NICM Health Research Institute, Western Sydney University, 158-160 Hawkesbury Road, Sydney, NSW 2145, Australia

**Keywords:** *Lepidium peruvianum*, *Serenoa repens*, COX-2, prostate cancer, benign prostate hyperplasia

## Abstract

Selected phenotypes of dried maca (*Lepidium peruvianum*) hypocotyls and supercritical CO_2_ extract (USPlus^®^) of saw palmetto (*Serenoa repens*) were used to determine their targeted, cytotoxic action in prostate cancer cells. Fingerprinting by HPLC-MS and PCA analysis showed compositional differences in glucosinolates, amides, macamides, and other alkaloids, which varied based on the color and the size of hypocotyls. These phytochemical differences translated into a higher antioxidant potential of red maca than black maca samples. The greatest COX-2 inhibition was demonstrated with a combination of red maca: saw palmetto (67%:33%) and red maca: saw palmetto: black maca (25%:50%:25%). The LNCaP androgen-dependent prostate cancer cell line was the most sensitive to the three-component mixture of black, red maca, and saw palmetto treatment. This combination provided the most abundant set of high-activity metabolites, and is worthy of consideration in further clinical applications and future in-depth study.

## 1. Introduction

Prostate cancer (PCa) is the second most common type of non-skin solid cancer and the fifth leading cause of cancer-related death in men. In 2018 the number of PCa cases worldwide comprised 1.276 million, with 359 thousand deaths. If the current trend continues, it is estimated that the global PCa cases will rise to 2.3 million in 2040 [1]. PCa mostly affects men older than 55 years, with 65% of men over 65 and 25% of men older than 75% having the condition [2]. Current treatment strategies for PCa include an active surveillance at the initial stages, followed by surgery and androgen deprivation therapy (ADT). In most cases PCa responds to ADT initially, but within disease progression, the disease develops androgen independence in the subsequent 2 years. At this stage, PCa is connected with a poor prognosis and requires treatment with chemotherapy and other adjuvant therapies [3].

Epidemiological data on PCa suggests the critical role of nutrition, besides the role of genetic factors, in developing this disease [1]. The role of proper nutrition has also been implicated in the prevention of benign prostatic hyperplasia (BPH), a common, nonmalignant, age-related enlargement of the prostate affecting ~70% of men aged 70 years or over [4]. Several phytochemicals and solvent extracts of plants used in traditional medicine in India and China demonstrated promising potential in maintaining prostate health and preventing the development of BPH and PCa. Herbal products showed selective toxicity towards abnormal prostate cells in vitro and in animal-based experiments with minimal toxicity to normal cells, suggesting their therapeutic potential against BPH and PCa diseases [1,5]. The subject of this study was two plant species characteristic of the South and North Americas: maca (*Lepidium peruvianum* Chacón, synonym *Lepidium meyenii* Walpers) and saw palmetto (*Serenoa repens* (Bartram) J.K. Small) with potential application in maintaining prostate health.

Maca (*L. peruvianum*) is an annual herbaceous plant of the Brassicaceae family, native to the high plateaus of the Peruvian central Andes, between 4000 and 4500 m altitude [6]. Maca presents three major phenotypes, red, yellow and black, based on their hypocotyl and stem coloration [7]. Some reports characterizing maca grown in Carhuamayo, Junín, in the Peruvian highlands describe 13 to 17 colors of maca hypocotyls, ranging from white to black [8]. Recently published reports suggest that maca phenotypes of different colors are associated with distinct phytochemical composition and reported biological effects or medical targets for which these different types can be used [9,10]. Regarding maintaining prostate health and preventing prostate cancer development, red maca is the most promising phenotype [11,12]. Red maca tubers, but not yellow or black, significantly reduced the ventral size of the prostate in male rats following the administration of 2 g dried raw maca hypocotyls/kg body weight for 7 constitutive days. Serum testosterone or estradiol levels were not affected by any of the phenotypes of maca assessed. Red maca also prevented the prostate weight increase induced by testosterone enanthate (TE) treatment [11]. On the other hand, black maca (but not red or yellow) was shown to increase daily sperm production and epididymal sperm motility [12]. Aqueous and hydroalcoholic extracts from red maca reduced prostate weight in rats with prostatic hyperplasia. This activity seems to be related to the content of benzyl glucosinolate [13]. Red maca also showed the highest total glucosinolate content among other phenotypes [14]. Additionally, our previous studies revealed statistically significant differences in total and specific glucosinolate content between red and black maca hypocotyls of different sizes: in general, small hypocotyls (<10 g) contained significantly higher amounts of total glucosinolates than large (>10 g) hypocotyls [14,15].

The extracts obtained from the berries of *Serenoa repens* (Bartram) J.K. Small, an American dwarf palm, have traditionally been used for treating lower urinary tract symptoms (LUTS) associated with BPH. The lipidosterolic extract of *S. repens* is produced by extraction with either n-hexane, ethanol, or supercritical fluid extraction with CO_2_. The product is a complex mixture of numerous fatty acids (70–95%; mainly lauric, myristic, palmitic, oleic, and stearic acids and their ethyl esters) phytosterols, and glycerides [16]. The commercial extract of saw palmetto (Permixon^®^) demonstrated inhibition of 5α reductase-I and II, the enzymes that convert testosterone to the biologically active dihydrotestosterone (DHT). Unlike other 5α-reductase inhibitors, *S. repens* extract does not influence the secretion of prostate-specific antigen (PSA), a PCa marker commonly used for diagnostic purposes [17]. Anti-prostate cancer activity of *S. repens* fruit extracts was confirmed using both in vitro [18,19] and animal models [20].

Reports suggest that prostate cancer is frequently associated with a shift in the oxidant/antioxidant balance, resulting in increased oxidative stress [21,22]. Reactive oxygen species (ROS), such as hydroxyl radicals, superoxide anion, and hydrogen peroxides, are capable of inducing lipid peroxidation and genomic DNA damage. Progressive age-related DNA damage and higher accumulation of 8-oxo-2′-deoxyguanosine (8-OHdG), an oxidized nucleoside of DNA, has been markedly increased in prostate cancer clinical specimens, compared to benign tissue [23,24]. Increased oxidative stress is also characteristic of chronic inflammation, which causes premalignant and malignant changes in the prostate [25]. Therefore, therapies maintaining prostate health and preventing prostatic diseases should be based on compounds with antioxidant activity.

An interesting target for anti-prostate cancer therapies is cyclooxygenase (COX), a rate-limiting enzyme in prostaglandin (PG) biosynthesis [26]. COX exists in two isoforms commonly known as COX-1 and COX-2. Although both isoforms catalyze the same enzymatic reactions, significant differences exist between them. COX-1 is constitutively expressed in a majority of cells, regulating vascular homeostasis, water reabsorption, gastric acid secretion, platelet aggregation, and renal blood flow. COX-2 is an inducible pro-inflammatory enzyme. Aberrant or increased expression of COX-2 has been found in most cancers, including prostate cancer, and the compelling evidence from genetic and clinical studies indicates that COX-2 upregulation is one of the key steps in developing PCa and BPH [27,28,29,30,31,32,33,34]. Celecoxib, a selective COX-2 inhibitor, demonstrated promising results in the prevention and treatment of prostate cancer [35]. Other COX-2 inhibitors have also been revised regarding their anti-prostate cancer effects [36]. The extract from *S. repens* specifically inhibited COX-2 expression in human prostatic cancer cell lines 267B-1, BRFF-41T, and LNCaP [37], and primary human prostate cancer cell [38]. No data on the influence of *L. peruvianum* extracts on COX-2 expression and activity is available in the scientific literature to date.

This study aimed to investigate the in vitro cytotoxicity towards human epithelial and prostate cancer cells, antioxidant activity, and COX-2 inhibitory potential of *L. peruvianum* extracts obtained from small and large, red and black hypocotyls, alone or in combination with *S. repens* extracts, as a potential complementary therapy for maintaining prostate health and preventing the development of prostate cancer.

## 2. Results and Discussion

### 2.1. Screening of the In Vitro Cytotoxicity of Red and Black Maca Hypocotyl Extracts on Human Prostate Cancer and Noncancerous Prostate Epithelial Cell Lines

**Figure 1:**
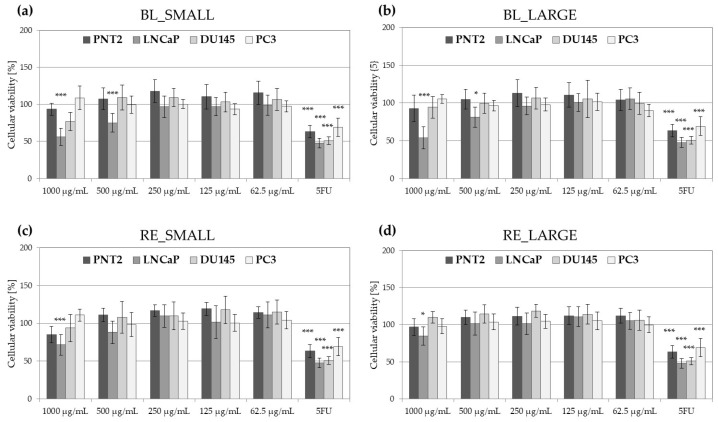
The viability of LNCaP, DU145, PC3 prostate cancer cell lines and noncancerous prostatic epithelial cells PNT2 following 48 h treatment with BL-SMALL (**a**), BL_LARGE (**b**), RE_SMALL (**c**), and RE-LARGE (**d**) extracts at 1000–62.5 µg/mL in comparison with 5 µg/mL 5-fluorouracil (5-FU); histograms show mean values ± SD, *n* = 15, *** *p* < 0.001, * *p* < 0.05.

Samples of small and large hypocotyls of black (BL_SMALL and BL_LARGE) and red (RE_SMALL and RE_LARGE) maca were compared for their cytotoxicity against three human prostate cancer cell lines: LNCaP (androgen-sensitive human prostate adenocarcinoma cells derived from the lymph node metastasis), DU145 (primary prostate adenocarcinoma derived from a central nervous system metastasis), and PC3 (prostate cancer cell line derived from bone metastasis). Human prostate epithelial cell line PNT2 was used as a non-cancer control. The cytotoxic effect of maca extract was compared with the commonly used chemotherapeutic agent 5-fluorouracil (5-FU) (Figure 1).

Black maca hypocotyl samples were more cytotoxic for analyzed prostate cancer cell lines, significantly decreasing the viability of LNCaP (at 1000 and 500 µg/mL) and DU145 (at 1000 µg/mL) cell lines. Red maca hypocotyl samples were cytotoxic only for LNCaP cells; however, they reduced cellular viability only by a max. of 30% at the highest-tested concentration. The LNCaP cell line was the most sensitive to maca samples treatment—the viability of cells following 48 h treatment with BL_SMALL extract was comparable with 5-FU. None of the tested maca samples was found to be cytotoxic for PNT2 cells.

In vitro cytotoxicity of maca extracts towards human prostate cancer cell lines was previously examined by Díaz and co-workers, who tested aqueous extracts from red maca collected in Peru. In this study, red maca extract at the concentration range from 10–80 µg/mL was not cytotoxic for LNCaP cells following 24 h and 48 h treatment but increased mRNA levels for androgen receptor (AR) and prostate-specific antigen (PSA) in these cells [39]. To our knowledge, no previously published studies have compared the in vitro cytotoxic activity of red and black maca hypocotyls of different sizes to human prostate cancer cell lines.

### 2.2. Screening of the In Vitro Cytotoxicity of Saw Palmetto Extracts on Human Prostate Cancer and Noncancerous Epithelial Cell Lines

**Figure 2:**
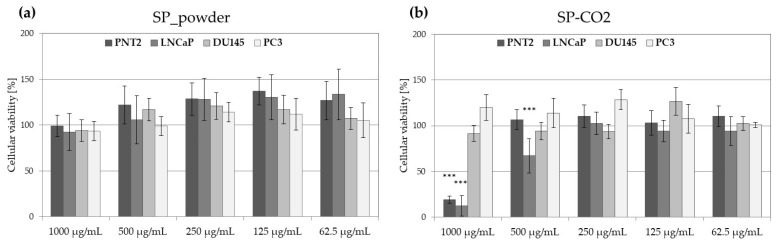
The viability of LNCaP, DU145, and PC3 prostate cancer cell lines and noncancerous prostatic epithelial cells PNT2 following 48 h of treatment with saw palmetto powdered extract (SP_powder) (**a**) or liquid extract obtained by supercritical CO_2_ extraction (SP_CO2) (**b**) at 1000–62.5 µg/mL; histograms show mean values ± SD, *n* = 15. A significant difference in cellular viability from control cells (100% viability) is marked with *** *p* < 0.001.

Saw palmetto extracts SP_powder and SP_CO2 samples were analyzed for their anti-prostate cancer cytotoxicity following 48 h of treatment (Figure 2). The SP_powder sample was not cytotoxic for analyzed prostate cells lines at all tested concentrations (Figure 2a), but the SP_CO2 sample was found cytotoxic for PNT2 and LNCaP cells at the highest tested concentration (1000 µg/mL), reducing the number of viable cells by 81–88%. At 500 µg/mL, the SP_CO2 sample was cytotoxic only against LNCaP cells, decreasing the cellular viability by 33% (Figure 2b).

**Figure 3:**
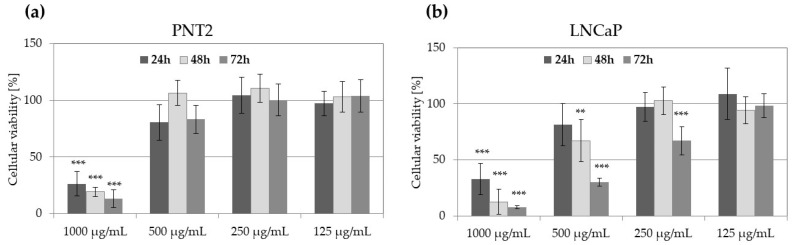
The viability of PNT2 (**a**) and LNCaP (**b**) cell lines following 24 h, 48 h, and 72 h treatment with various concentrations of saw palmetto liquid extract obtained by supercritical CO_2_ extraction (SP_CO2); histograms show mean values ± SD, *n* = 15. A significant difference in cellular viability from control cells (100% viability) is marked with ** (*p* < 0.01) or *** (*p* < 0.001).

To further explore the influence of SP_CO2 extract on PNT2 and LNCaP, the viability of cells was compared following 24 h, 48 h, and 72 h extract treatment (Figure 3). Obtained results indicate that the cytotoxic effect of the SP_CO2 sample against LNCaP cells increases with the time of the treatment.

In the presented study, a cytotoxic effect of *S. repens* extract was detected only towards the LNCaP prostate cancer cell line. However, based on the previously published data, it might be concluded that the extracts from *S. repens* may affect both androgen-sensitive and androgen-insensitive PCa cells. Ethanol fruit extract of *S. repens* showed significant in vitro toxicity toward androgen-sensitive LNCaP cells and induced apoptosis in a concentration-dependent manner. The GI50 value (the concentration of the agent that inhibits the growth by 50%), established by WST-1 assay or CV staining, was 127.7 ± 17.5 µg/mL and 195 ± 7.1 µg/mL, respectively. Androgen-insensitive DU145 cells were less sensitive to *S. repens* extract treatment [18]. At 1–10 µg/mL, *S. repens* extract inhibited the invasion of the androgen-insensitive PC3 prostate cancer cell line in the Transwell migration assay through the inhibition of the urokinase-type plasminogen activator (uPA). The invasive properties of LNCaP cells, expressing less uPA, were not affected by *S. repens* extract [19]. Yan et al. showed that *S. repens* extract induced the growth arrest of prostate cancer LNCaP, DU145, and PC3 cells with ED50s of approximately 2.0, 2.6, and 3.3 µL/mL, respectively. The decreased viability of LNCaP cells was induced by *S. repens* extract due to apoptosis and involved inactivation of STAT3 and androgen receptor signaling pathways [40]. The studies by Opoku-Acheampong et al. indicated that fatty acids and phytosterols present in *S. repens* supplements were responsible for the cytotoxic effect of the extract [20]. Iguchi and co-workers identified myristoleic acid as the cytotoxic compound of *S. repens* extract, inducing apoptosis and necrosis in LNCaP cells in a dose-dependent manner [41].

### 2.3. Screening of the In Vitro Cytotoxicity of Maca and Saw Palmetto Combinations on Human Prostate Cancer and Noncancerous Epithelial Cell Lines

In subsequent experiments, the most cytotoxic SP_CO2 extract was mixed with BL_SMALL, BL_LARGE, RE_SMALL, or RE_LARGE extracts and tested for its cytotoxicity against PNT2 and LNCaP cells. The obtained data and the combination of tested samples are summarized in Table 1. The viability of LNCaP cells treated with BL-SMALL, BL-LARGE samples alone or in combination with SP_CO2 extracts was significantly reduced in comparison with control, solvent-treated cells. However, no significant differences in cellular toxicity were observed between maca samples and the combinations of maca with saw palmetto.

### 2.4. Antioxidant and Anti-Inflammatory Activity of Maca and Saw Palmetto Extracts

**Figure 4:**
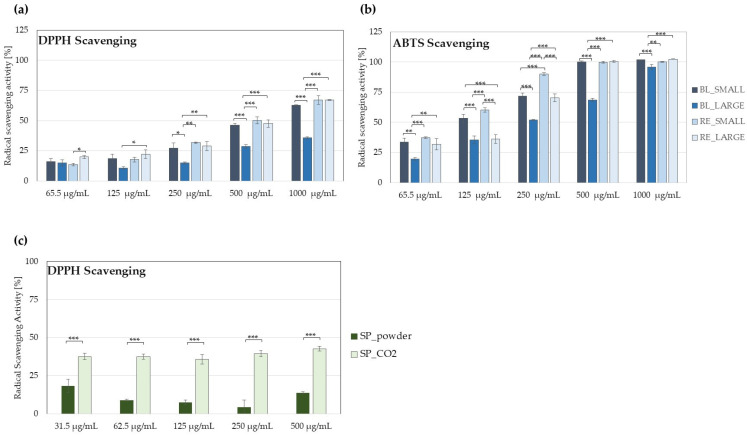
DPPH (**a**,**c**) and ABTS (**b**) radicals scavenging activity of maca (**a**,**b**) extracts at 1000–62.5 µg/mL and saw palmetto extracts (**c**) at 31.25–500 µg/mL; histograms show mean values ± SD, *n* = 3 (BL_LARGE—black large hypocotyls, BL_SMALL black small hypocotyls, RE_LARGE—red large hypocotyls, RE_SMALL—red small hypocotyls); * *p* < 0.05, ** *p* < 0.01, *** *p* < 0.001.

Nutrients with significant antioxidant activity are important in the prevention of various types of cancers, including prostate cancer. Therefore, the antioxidant potential of red and black maca extracts prepared from small and large tubers was compared using DPPH and ABTS scavenging assay. As presented in Figure 4a,b, RE_SMALL and RE_LARGE extracts showed significant antioxidant potential. Regarding black maca, the BL_SMALL sample was significantly more active than the BL_LARGE extract. The antioxidant activity was assessed also for SP_powder and SP_CO2 samples; in DPPH radical scavenging assay, the SP_CO2 extract showed more significant antioxidant potential (Figure 4c).

In order to gain a deeper understanding of the impact of maca and saw palmetto extracts on prostate cancer cells biology, the COX-2 inhibitory activity was tested. Targeting COX-2 was shown to significantly reduce the growth of prostate cancer cells in vitro and in animal models and supports the therapy of prostate hyperplasia [42,43]. As can be seen in Table 2, RE_SMALL extract used singly (samples 1, 2, and 3) showed a moderate ability to reduce COX-2 activity in a dose-dependent manner (48.5 ± 3.7% reduction at the highest-tested concentration). To improve the effect, we first tested the most efficient combinations of maca components in terms of the anti- cancer and antioxidant activities (RE_LARGE and BL_SMALL). In this way, sample 4 was created (SP_powder 33% + RE_LARGE 67%), which resulted in a very high ability to reduce COX-2 activity compared to RE_LARGE extract applied singly. Similarly, the introduction of BL_SMALL (sample 5: SP powder extract 50% + RE_LARGE and BL_SMALL extracts, both at 25%) gave the same effect (Table 2). A change in a saw SP powder:RE_LARGE:BL_SMALL ratio (samples 6 and 7) and the removal of BL_SMALL from the mixture (samples 8, 9, and 10), resulted in a slight decrease in anti-COX-2 activity. These findings indicate the occurrence of synergism between the three components of the mixture and the need for a specific selection of the proportions of the three components of the plant mixture with respect to the maca phenotype, hypocotyl size, and cultivation location, to obtain the maximum desired effect.

### 2.5. The Fingerprinting of the Investigated Maca Extracts by the HPLC-MS Approach

**Figure 5:**
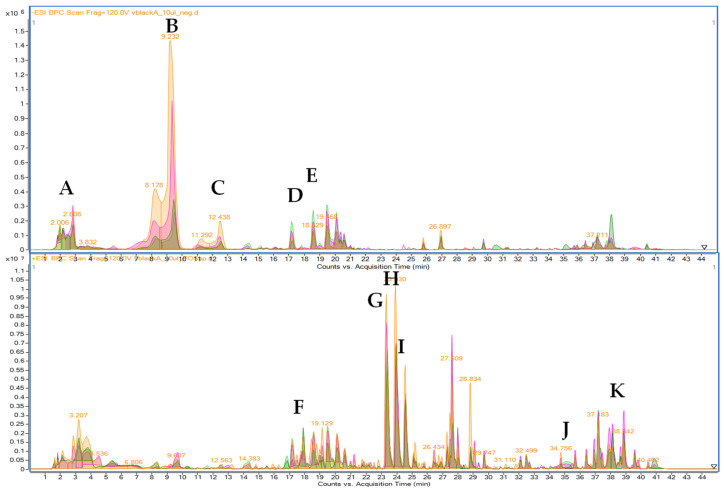
The fingerprints of the analyzed extracts recorded in the negative (**above**) and positive (**below**) ionization mode of HPLC-MS analysis (The letters A–K represent the tentatively identified compounds as in the Table 3).

The extracts from small and large hypocotyls of both red and black maca were injected on HPLC-ESI-QTOF-MS/MS in both positive and negative ionization modes. The performed HPLC-MS-based analyses let us notice the rich fingerprint of the analyzed extracts. The hypocotyls of *Lepidium peruvianum* species and its different phenotypes are rich sources of secondary metabolites of different types. The negative mode provided evidence of the presence of glucosinolanes (Figure S1 in the Supplementary File, Figure 5, Table 3), whereas the positive operation mode revealed the relations in the content of alkaloids, amides, and other components characteristic of the maca extract (Figure S2 in the Supplementary File, Figure 5, Table 3). The total activity of the extracts is certainly induced by different classes of metabolites that possibly exert synergistic interactions.

The compositional analysis of maca tuber is still a large challenge to scientists as the plant produces very specific components. Also, as a plant rich in glucosinolanes and their decomposition products, the fingerprints may differ, and the single signals may be hard to be tentatively identified based on the HPLC-MS profile and without isolation.

A few scientific papers have tried to assign the recorded *m*/*z* features to chemical structures. Among them, Tarabasz et al. [15] focus on the glucosinolanes present in the tubers, McCollom et al. [44] discuss the presence of macamides in the plant, whereas Jin and collaborators underline the significance of lepidilines as the leading components of maca [45]. As mentioned above, the number of metabolites in the species is tremendous, as confirmed by an extensive HPLC-MS-based fingerprinting published by Zhou and co-investigators [46]. However, in this manuscript, we decided to focus on the tentative identification of several representatives of the different classes of metabolites that are present in the herein-investigated extracts in the highest quantity. Table 2 presents the results of a tentative assignment of some representatives of glucosinolanes, lepidilines, macamides, and amides. Among them, the leading components of the fingerprint recorded in the negative ion mode were glucotropaeolin—the major glucosinolane of the extract followed by glucolimnanthin. The positive ion mode was dominated by lepidilines D > E > A > C. However, macamide B and a benzyl-oxo-octadienamide derivative were also present in marked quantities.

**Figure 6:**
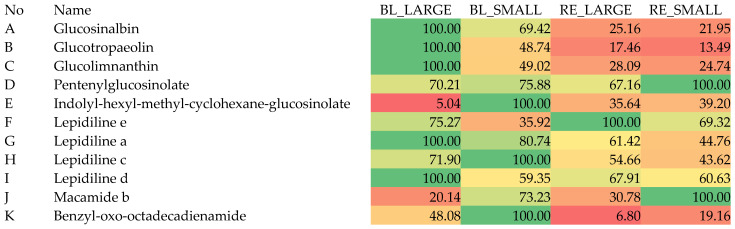
The relative percentage content of 11 metabolites tentatively identified in this study (the highest peak are of each component was taken as 100% and is marked as green, and the following contents in one row are calculated in relevance to the highest peak area with red-coloured fields indicating the smallest content).

Figure 6 below shows their relative content data that can be analyzed in the context of biological studies. The peak areas of the 11 metabolites were collected from the four extracts (*n* = 3) and averaged. The largest peak area was taken as 100%, and the remaining peak areas were calculated in relation to the highest peak, for every compound separately. Figure 6 below presents the differences in the content of the tentatively identified components. The obtained data show a different profile of black tubers of maca compared with the red ones. This small study provides proof that these different phenotypes may indeed carry different pharmacological potential based on their differentiated composition. Similar conclusions were drawn in the previous studies performed by our team and also by other authors [10,15].

### 2.6. PCA and Clustering Analysis of Black and Red Maca Samples

**Figure 7:**
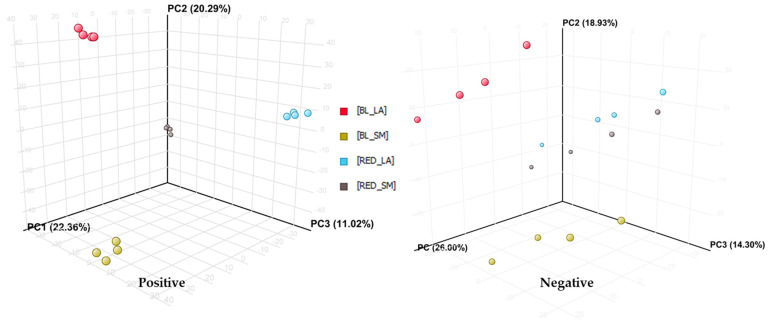
The results of principal component analysis (PCA) with the scores obtained for the first three principal components (BL_LA—black large hypocotyls, BL_SM—black small hypocotyls, RE_LA—red large hypocotyls, RE_SM—red small hypocotyls).

As shown in Figure 7, black phenotypes of maca were found to be richer in the analyzed leading molecules of the fingerprints. However, the plentitude of signals recorded in both ion modes during the HPLC-MS analysis encouraged the authors to perform a deeper metabolomic analysis of maca tuber extracts to search for other molecular features that may play an important role in the differentiation of the samples.

As a result of principal component analysis (PCA), three components (PC) were distinguished that together explained 53.67% of the variation (22.36%, 20.29%, and 11.02% for PC1, PC2, and PC3, respectively) for the mass chromatograms recorded in the positive ionization mode, and 59.23% of the variation (26.00%, 18.93%, and 14.30% for PC1, PC2, and PC3, respectively) for the mass chromatograms in the negative ion mode (Figure 7).

The performed analysis shows a high differentiation of the results obtained for every studied extract for the mass chromatograms recorded in the positive ionization mode. Certainly, the color and size of the maca phenotype determine its composition.

For mass chromatograms recorded in the negative ionization mode, it was noted that red large hypocotyls and red small hypocotyls are similar in the content of compounds of this nature. In addition, large black hypocotyls are significantly differentiated from the others similarly to red small hypocotyls.

The cluster analysis results yield similar results for both the mass chromatograms recorded in the positive and negative ionization modes. In both cases, two clusters can be distinguished: one containing extracts from BL_LARGE tubers and BL_SMALL tubers, the other containing extracts from red large tubers and red small tubers.

**Figure 8:**
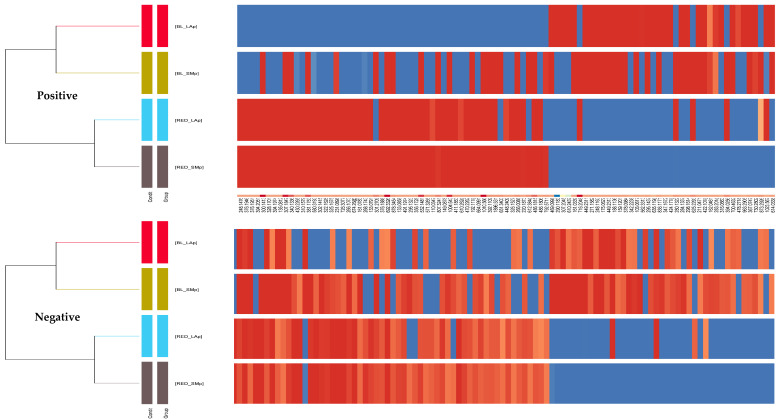
Heat maps of maca extracts with the results of hierarchical analysis showing the intensity of contained features (red: high content, blue: low content) when analyzing the molecular features present in the recorded mass chromatograms recorded in the positive and negative ionization mode.

Below, the graphs from the hierarchical analysis show the sample BL_LARGE and RE_SMALL bear opposite properties (Figure 8).

Since BL_LARGE and RE_SMALL were the most active, we searched for such compounds whose FC will be similar for these two samples and at the same time will be strongly different from the others, i.e., BL_SMALL and RE_LARGE. To deliver more precise information about the character of differences among the samples, ANOVA statistical analysis enabled a direct comparison of the composition of the two varieties that exhibited the most advantageous pharmacological properties (BL_LARGE and RE_SMALL)—with other extracts. The *m*/*z* signals that were differentiating the samples were also discovered. The *m*/*z* signals differentiating the two most active extracts from the rest of the tested samples were: in the negative mode: 550.1819, 943.5956, 723.2300, 877.4493, 664.2240; in the positive mode: 716.2048, 873.2926, 625.2293, 394.256, 298.1322.

**Figure 9:**
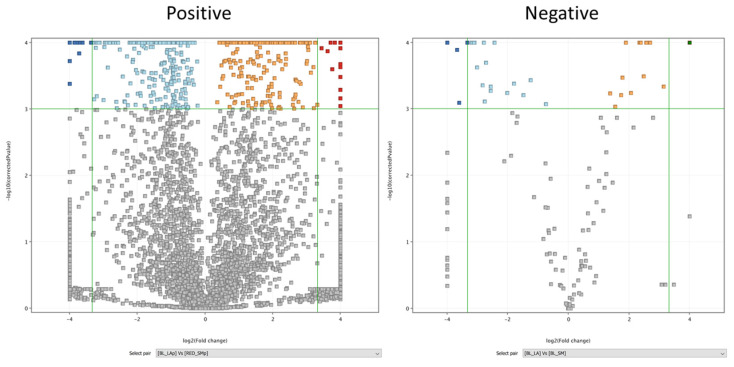
The volcano plots for the data from the positive and negative ionization of the tested samples: BL_LARGE vs. RE_SMALL.

In addition, the compositions of these two extracts were compared. The results of these analyses are shown in volcano-type graphs (Figure 9), where the following cutoff points were used: Fold Change (FC) ± 10 and *p*-value ≤ 0.001.

The volcano plot enabled the discovery of these differentiating signals, as shown in the pictures below in the top right and top left corners. As presented in the figure below, the differences between the two samples were higher in the positive ionization mode (22 with negative FC and 9 with positive FC in the negative ionization mode vs. 112 with negative FC and 125 with positive FC in the positive ionization mode). The differentiating signals were presented in the Supplementary File (Table S2).

## 3. Materials and Methods

### 3.1. Chemicals and Reagents

5-fluorouracil (5-FU), Dulbecco’s phosphate-buffered saline (DPBS), Dimethyl sulfoxide (DMSO), 2,2-Diphenyl-1-picrylhydrazyl (DPPH), 2,2′-Azino-bis(3-ethylbenzothiazoline-6-sulfonic acid) diammonium salt (ABTS), 3.3 g/L Neutral Red solution in DPBS, cell culture media, and other reagents required for cell culture (L-glutamine, trypsin-EDTA solution) were purchased from Sigma-Aldrich (Merck Group, Darmstadt, Germany). Fetal bovine serum (FBS) was obtained from Pan-Biotech (Aidenbach, Germany). Ethanol (>98%) and glacial acetic acid were purchased from Honeywell (Charlotte, NC, USA). Standards for HPLC-MS fingerprinting, namely, glucotropaeolin, glucolimnanthin, and glucosinalbin, were purchased from Extrasynthese (Genay, France), macamide B and lepidiline B were obtained from ChemFaces (Wuhan, China).

### 3.2. Maca Hypocotyls and Saw Palmetto Extracts

Maca samples were obtained from the dried and pulverized hypocotyls of *Lepidium peruvianum* collected in the highlands of Peru—in the Junín Plateau or the Ancash region. Freshly collected maca hypocotyls were separated from one another according to their color, size, and cultivation site, and transported to an open-air drying location. After a traditional drying system, they were pulverized and stored in a cool, dry place. The following types of maca hypocotyls were used: red maca small hypocotyls—**RE_SMALL** and red maca large hypocotyls (**RE_LARGE**) were obtained from the Ancash region in Cordillera Blanca in the Peruvian Andes (AN-17) in mid-October 2017, whereas black maca large hypocotyls (**BL_LARGE**) and black maca small hypocotyls (**BL_SMALL**) were collected in Junín (J-19) Maca plantation on 8 October 2019 between 11 am and 2 pm local time. Black maca samples (Large and Small) were also collected in the Ancash plantation region. All the maca hypocotyl samples were later processed in a commercial operation in Lima, before being transported by an air courier to the laboratory at the Medical University of Lublin, where they were subjected to a laboratory-scale extraction procedure. Water: ethanol extracts (50:50; *v*/*v*) were obtained from the powdered plant material using ultrasound-assisted extractor (UAE). For this purpose, 1 g of powdered hypocotyls was sonicated with 10 mL of the extracting solvent for 30 min. The sample was then centrifuged at 10,000 rpm, and the supernatant was transferred to a clean “falcon” tube. The remaining plant material was soaked in the new portion of the extracting solvent (5 mL), and the extraction was repeated. The supernatants were combined and evaporated to dryness at 45 °C using a centrifuge with a vacuum (Eppendorf Concentrator plus, Hamburg, Germany). The obtained dried residues were stored at −20 °C until further testing. In the experiments presented in this paper, two types of saw palmetto extracts (*Serenoa repens*–*Sabal serrulata*) were obtained from the company Valensa International (2751 Nutra Ln, Eustis, FL 32726, USA). One of them (**SP_CO2**) was obtained by the super-critical CO_2_ extraction technique USPlus^®^, permitted in the EU and US Pharmacopeia for Saw Palmetto [47,48]. Extraction was performed using a patented Supercritical Fluid CO_2_ technique performed in a commercial extraction operation with the dried berry: oil extract ratio 7–8:1. The other, dry extract of Saw Palmetto (**SP_powder**) was prepared under the same extraction conditions as SP_CO_2_ using a water: ethanol mixture (50:50; *v*/*v*) and UAE.

### 3.3. Antioxidant Activity Assays

#### 3.3.1. DPPH Radical Scavenging Assay

Antiradical activity of maca samples was established using a DPPH scavenging assay described by Matejic et al. [49] with further modifications [50]. Briefly, 100 μL of DPPH working solution (25 mM in 99.9% methanol; A_540_ ≈ 1) was mixed with an equal volume of maca and saw palmetto samples diluted in 96% (*v*/*v*) ethanol (62.5–1000 µg/mL) or the solvent as a control sample, followed by 20 min incubation at room temperature in darkness. The absorbance of the samples was measured at λ = 540 nm using a FilterMax F5 microplate reader (Molecular Devices, San Jose, CA, USA), and the obtained values were used to calculate the percentage of DPPH radical scavenging based on the following equation:

% of DPPH˙ scavenging = [1 − (Abs(S)/Abs(C))] × 100% (1)

where: Abs(S)—the absorbance of the sample, Abs(C)—the absorbance of the control sample.

#### 3.3.2. ABTS Radical Scavenging Assay

The ABTS radical scavenging assay was performed according to the protocol described by Re and co-workers [51] with further modifications [50]. 15 μL of maca samples (62.5–1000 µg/mL) was mixed with 135 μL ABTS working solution (7 mM ABTS in 2.45 mM K_2_S_2_O_8_ diluted in distilled H_2_O to A_405_ ≈ 1) and incubated for 15 min at room temperature in darkness. Equal volume of H_2_O mixed with ABTS working solution was used as a control sample (100% radical activity). The absorbance of the samples was measured at λ = 405 nm using a microplate reader (FilterMax F5 Molecular Devices), and the obtained values were used to calculate the percentage of ABTS radical scavenging based on the following equation:

% of ABTS scavenging = [1 − (Abs(S)/Abs(C))] × 100(2)

where: Abs(S)—the absorbance of the sample, Abs(C)—the absorbance of the control sample.

### 3.4. Inhibition of COX-2

Dried extracts of maca and saw palmetto (prepared using water: ethanol (50:50)/UAE extraction as described in Section 3.2) were dissolved in DMSO (12.5, 25 or 50 mg/mL of red maca and 50 mg/mL in the case of two other extracts). The analysis was performed exactly as described in Kukula-Koch et al. [52] except the following volumes of reagents were used in the reaction mixture: 0.03 mL tested sample, 0.1 mL Tris–HCl buffer, 10 mL of hemin, 0.02 mL colorimetric substrate solution, 0.02 mL arachidonic acid solution, and 0.02 mL COX-2 enzyme solution.

### 3.5. In Vitro Cytotoxicity

#### 3.5.1. Cell Lines

Human prostate cancer cell lines LNCaP, DU145, and PC3 as well as prostate epithelial cell line PNT2 were kindly provided by Dr Vera Knäuper, School of Dentistry, Cardiff University (Cardiff, UK). LNCaP and PNT2 cell lines were maintained in Roswell Park Memorial Institute (RPMI) 1640 medium, PC3 was grown in Ham’s F-12 Nutrient Mixture, and DU145 cells were grown in Eagle’s Minimum Essential Medium (EMEM). All culture media were supplemented with 10% *(v*/*v)* FBS. The cells were cultured at 37 °C in a humidified atmosphere with 5% CO_2_.

#### 3.5.2. Neutral Red Uptake Test

In vitro cytotoxicity of maca and saw palmetto extracts compared to 5-FU was analyzed using the Neutral Red Uptake Test described by Repetto and co-workers [53]. Each experiment was performed using 96-well plates, with 3 × 10^3^ cells per well. The cells were grown overnight and then treated with various concentrations of maca or saw palmetto samples (62.5–1000 µg/mL) dissolved in DMSO or 5 µg/mL 5-FU (positive control). Negative control cells (100% viability) were grown in an appropriate culture medium containing an equal volume of DMSO. Following 48 h of culture the cells were incubated for 3 h with 33 µg/mL neutral red solution in a conditioned medium supplemented with 1% FBS, then rinsed with DPBS and lysed using an acidified ethanol solution (50% *v*/*v* C_2_H_5_OH, 1% *v*/*v* CH_3_COOH, and 49% H_2_O). The absorbance of the released neutral red dye was measured spectrophotometrically using a FilterMax F5 microplate reader (Molecular Devices, San Jose, CA, USA) at λ = 540 nm and corrected by the absorbance measured at λ = 620 nm. The mean measured value for the lysate from control cells was set as 100% cellular viability and used to calculate the percentage of viable cells following each sample treatment. Each experiment was performed three times with five repetitions for each tested concentration (*n* = 15). Cytotoxicity experiments on combined maca and saw palmetto samples were performed twice, with five repetitions per condition (*n* = 10). Mean and standard deviation (SD) values were calculated using Microsoft Excel.

### 3.6. Fingerprinting of Maca Extracts by the HPLC-ESI-QTOF-MS/MS Platform

The fingerprints of the tested extracts were recorded by the HPLC-ESI-QTOF-MS/MS (a high-performance liquid chromatograph with an electrospray ionization source, a QTOF analyzer, and the MS/MS fragmentation possibility) platform in both positive and negative ionization mode. Before the analysis, the dried residue was dissolved in a mixture of ethanol and water (50:50 *v*/*v*) at a concentration of 10 mg/mL. Later, the mixture was filtered through a nylon syringe filter (0.1 µm pore size) and subjected to HPLC-MS analysis using an Agilent Technologies instrument composed of a 1200 Series HPLC chromatograph coupled with ESI-QTOF-MS/MS spectrometer (G6530B). The chromatographic separation was achieved on the chromatographic column–Zorbax Eclipse Plus RP18 (150 × 2.1 mm; dp = 3.5 µm) by Agilent Technologies (Santa Clara, CA, USA) in the following gradient of ACN with 0.1% formic acid in 0.1% aqueous solution of formic acid: 0 min 1: 99%, 4 min 20: 80%, 20 min 60: 40%, 35 min 95: 5%, 38 min 95: 5%, 39 min 1: 99%. The analysis lasted for 45 min, the flow rate was set at 0.2 mL/min, the temperature at 25 °C, and the injection volume at 10 µL. The following settings of the mass spectrometer were applied: the capillary voltage of 3500 V, the gas flow of 12 L/min, the gas temperatures of 350 and 325 °C for gas and sheath gas, respectively, the nebulizer pressure of 35 psig, the skimmer voltage of 65 V, the fragmentation energy of 120 V, and the collision energy of 10 and 20 V. The spectra were collected for the *m*/*z* range of 100–1700 Da in two ionization modes—positive and negative ions. The recorded spectra are presented in the Supplementary Materials (Figures S1 and S2). The data were analyzed by the Mass Hunter Qualitative Analysis Navigator (version B.10.00, Agilent Technologies) and by Mass Profiler Professional (version 15.1, Agilent Technologies). The tentative identification of the compounds was performed based on the scientific literature, retention times, and the mass spectrometry databases (Metlin, Human Metabolome Database). A few of the assigned compounds were analyzed by the comparison with the authentic standards, which was marked in the Table 2. The identification of lepidilines was based on the injection of lepidiline B standard and a comparison of the fragmentation patterns of the remaining components.

### 3.7. Statistical Analysis

The statistical significance of the biological activities between the analyzed samples was analyzed using GraphPad Prism 7.0 Software (GraphPad Software, San Diego, CA, USA). Results obtained for different samples were analyzed using one-way ANOVA and Tukey’s test. All results presented on graphs represent mean ± SD.

The chemometric studies conducted using the HPLC-MS data were recorded in the Mass Profiler Professional Software (version 15.1-build 15.1.20045.0 by Agilent Technologies Inc., Santa Clara, CA, USA). The PCA analysis and cluster analysis were performed at *p* < 0.01 using 1763 entities in the positive ion spectra and 620 entities in the negative ion spectra. The comparison was performed using the one-way ANOVA with Multiple Testing Correction in the Benjamini–Hochberg test.

## 4. Conclusions

The LNCaP androgen-dependent prostate cancer cell line was the most sensitive to maca and saw palmetto treatment among all the tested prostate cell lines. Black maca samples (BL_SMALL and BL_LARGE) were more active than red maca extracts. Among the red maca samples, RE_SMALL was more cytotoxic than RE_LARGE. The samples obtained from saw palmetto fruit using CO_2_ supercritical extraction (SP_CO2) were significantly more active than the extracts prepared using UAE, and their cytotoxicity increased with the treatment time. Combination of red or black maca with the SP_CO2 sample did not significantly increase the cytotoxic effect towards LNCaP cells. Red maca samples showed higher antioxidant potential when compared with black maca extract samples. Regarding black maca, the BL_SMALL sample was significantly more active than the BL_LARGE sample. Interestingly, a synergistic effect on COX-2 inhibition was demonstrated with a combination of red maca:saw palmetto (67%:33%) and red maca:saw palmetto:black maca (25%:50%:25%).

The performed HPLC-MS-based fingerprinting showed that maca hypocotyls are rich sources of different types of metabolites, with rich representation of glucosinolanes in the negative ion mode and amides, saccharides, lepidilines, macamides, and other alkaloids in the positive ion mode. The performed PCA analysis on the molecular features extracted from the recorded mass chromatograms showed marked compositional differences between the analyzed samples in terms of the peak areas of the respective *m*/*z* signals; the obtained graphs showed that the color and size of hypocotyls differentiated the composition of every extract maca sample tested in this work. The performed statistical analysis of samples provided a list of *m*/*z* signals discriminating the samples. Interestingly, large black and small red tubers were characterized by opposite features that were pictured in Figure 8 from the hierarchical analysis; possibly, when mixed together in preparation, the combined red small, Ancash-cultivated, and black large Junín cultivated hypocotyls provided the richest set of metabolites with different biological properties that determined the total activity of the mixture.

## 5. Patents

The data presented in this manuscript are included in the patent application P.449199 submitted to the Patent Office of the Republic of Poland on 7 July 2024. The title of the patent application is “The composition of plant extracts from Peruvian Maca (*Lepidium peruvianum*) and Saw Palmetto (*Serenoa repens*) supporting prevention and therapy of prostatic hyperplasia and application of the composition of plant extracts from Peruvian Maca (*Lepidium peruvianum*) and Saw Palmetto (*Serenoa repens*) supporting the prevention and therapy of prostatic hyperplasia”.

## Figures and Tables

**Table 1:** Cytotoxic activity of combined maca and saw palmetto samples following 48 h treatment of human prostate epithelial cells PNT2 and prostate cancer cells LNCaP; cell viability values were calculated in comparison with control, solvent-treated cells (100% viability) from two independent experiments with five repetitions per condition (*n* = 10). A significant difference in cellular viability from control cells (100% viability) is marked with ** (*p* < 0.01) or *** (*p* < 0.001).

Sample No.	Maca Extract	SP_CO2	PNT2 Viability (in %; Mean ± SD)	LNCaP Viability (in %; Mean ± SD)
1	-	0.33 mg/mL	109.08 ± 16.43	83.65 ± 17.66
2	BL_SMALL 0.67 mg/mL	-	95.78 ± 16.43	59.78 ± 16.79 **
3	BL_LARGE 0.67 mg/mL	-	114.97 ± 12.66	58.14 ± 15.11 **
4	RE_SMALL 0.67 mg/mL	-	117.25 ± 12.88	74.84 ± 15.13
5	RE_LARGE 0.67 mg/mL	-	111.84 ± 8.00	69.07 ± 11.25
6	BL_SMALL 0.67 mg/mL	0.33 mg/mL	88.72 ± 11.35	50.06 ± 15.26 ***
7	BL_LARGE 0.67 mg/mL	0.33 mg/mL	92.18 ± 8.88	56.27 ± 12.00 **
8	RE_SMALL 0.67 mg/mL	0.33 mg/mL	95.18 ± 8.88	71.41 ± 22.78
9	RE_LARGE 0.67 mg/mL	0.33 mg/mL	100.72 ± 9.44	76.81 ± 19.27

BL_LARGE—black large hypocotyls, BL_SMALL—black small hypocotyls, RE_LARGE—red large hypocotyls, RE_SMALL—red small hypocotyls, SP_CO2—saw palmetto supercritical CO_2_ extract.

**Table 2:** Inhibition of COX-2 by maca and saw palmetto extracts.

	% Content in the Reagent Solution (μg of the Extract in the Well)			**% Inhibition** **of COX-2 ± SD**
No	SP_Powder	RE_LARGE	BL_SMALL	
1	-	100 (375)	-	6.1 ± 1.2
2	-	100 (750)	-	24.2 ± 0.3
3	-	100 (1500)	-	48.5 ± 3.7
4	33 (500)	67 (1000)	-	87.9 ± 2.8
5	50 (750)	25 (375)	25 (375)	87.9 ± 1.0
6	20 (300)	60 (900)	20 (300)	81.8 ± 4.9
7	30 (450)	55 (825)	15 (225)	78.8 ± 3.3
8	50 (750)	50 (750)	-	81.8 ± 4.7
9	75 (1125)	25 (375)	-	81.8 ± 5.6
10	15 (225)	85 (1275)	-	78.8 ± 4.1

RE_LARGE—red large hypocotyls, BL_SMALL—black small hypocotyls, COX-2—cyclooxygenase-2.

**Table 3:** The tentative identification of the major metabolites present in the negative and positive ion mode in the analyzed extracts from *Lepidium peruvianum* (DBE—number of double bonds and rings, ion—ionization mode, *—the identification was performed based on the comparison with an authentic standard).

No	Ion.+/−	Rt [min]	Molecular Formula	*m*/*z* Theoretical	*m*/*z* Experimental	Error (ppm)	DBE	MS/MS Spectrum	Proposed Compound
A	−	5.54	C_14_H_19_NO_10_S_2_	424.0378	424.0388	−2.44	6	328.0038; 259.0062; 182.0237	Glucosinalbin *
B	−	9.3	C_14_H_19_NO_9_S_2_	408.0428	408.0446	−4.29	6	259.0023; 166.0271	Glucotropaeolin *
C	−	12.59	C_15_H_21_NO_10_S_2_	438.0534	438.0544	−2.25	6	259.0081; 196.0418	Glucolimnanthin *
D	−	17.13	C_12_H_24_O_7_N_2_S_2_	371.0952	371.0978	−6.94	2	249.0626	Pentenylglucosinolate
E	−	18.47	C_22_H_38_O_9_N_2_S_2_	521.1997	521.2031	−6.55	5	359.1562; 329.1425	Indolyl-hexyl-methyl-cyclohexane-glucosinolate
F	+	17.9	C_15_H_18_N_2_	227.1543	227.1538	2.1	8	135.0876	Lepidiline E
G	+	23.45	C_19_H_20_N_2_	277.1699	277.1684	−4.18	6.5	185.1019	Lepidiline A
H	+	23.97	C_20_H_22_N_2_O	307.1805	307.1791	4.54	11	171.0788	Lepidiline C
I	+	24.61	C_21_H_24_N_2_O	321.1961	321.1946	4.81	11	230.1108 199.0941	Lepidiline D
J	+	35.03	C_23_H_39_NO	346.3104	346.3070	2.2	0.5	308.1678	Macamide B *
K	+	38.88	C_25_H_37_NO_2_	384.2906	384.2877	5.23	8	260.1876	Benzyl-oxo- octadecadienamide

## Data Availability

The data sets presented in the current study are available from the corresponding authors upon reasonable request.

## Supplementary Materials

The following supporting information can be downloaded at: https://www.mdpi.com/article/10.3390/molecules29235632/s1, Figure S1: The total mass chromatograms recorded in the negative ionization mode for the tested maca samples; Figure S2: The total mass chromatograms recorded in the positive ionization mode for the tested maca samples; Table S1: Raw data used for the volcano-type graphs, where the following cut-off points were used: FC +/− 10 and *p* value ≤ 0.001; Table S2: The MS/MS spectra of the tentatively identified components of *Lepidium peruvianum* extracts; Table S3: Raw data used for the volcano-type graphs, where the following cut-off points were used: FC +/− 10 and *p* value ≤ 0.001.
