# Supplementary material for: Synergism of Specific Maca Phenotypes (Lepidium peruvianum) in Combination with Saw Palmetto (Serenoa repens) Extract for Chemoprevention of Prostate Cancer as Determined in In Vitro Cytotoxicity Assays on Human Epithelial and Prostate Cancer Cells
Source: Molecules. 2024 Nov 28;29(23):5632. doi: 10.3390/molecules29235632 (PMC11643235; doi:10.3390/molecules29235632)
Supplement: Supplementary file 1 [file molecules-29-05632-s001.zip › molecules-3308998-supplementary.pdf]

Table S1. Raw data used for the volcano-type graphs, where the following cut-off points were used: FC +/- 10 and p value ≤ 0.001.

Positive ionization mode			
**Compound**	FC (BL_LARGE vs RE_SMALL)	FC (BL_SMALL vs RE_SMALL)	FC (RE_LARGE vs RE_SMALL)
**332.1582@12.779002**	-351582.53	-351583	-351583
**183.1081@8.895998**	944966.1	193905.6	292198.9
**332.1821@15.537998**	1952130.2	1344371	1780623
**394.1679@2.1199994**	1346078	789117.2	796640.4
**340.1479@11.869999**	202920.9	347487.3	164694.1
**332.188@13.166**	151019	361437.9	376447
**708.406@14.731998**	44953.305	2016271	391480
**637.1181@8.558999**	49217.72	335920.8	99725.36
**537.2063@9.862**	65774.266	122407.1	117900.9
**642.267@12.203002**	89673.41	37662.87	17886.46
**282.4973@11.561998**	118518.72	192161	31734.24
**250.1342@13.998**	165751.36	69259.63	49082.79

Negative ionization mode			
**Compound**	FC (BL_LARGE vs RE_SMALL)	FC (BL_SMALL vs RE_SMALL)	FC (RE_LARGE vs RE_SMALL)
**463.2547@10.4470005**	14152.336	9173.428	-10.7329
**876.1531@14.194**	-1057.2604	-1057.2604	26.2196
**846.1342@14.11**	-1108.4723	-1108.4723	33.48628
**333.0914@10.916**	-1174.285	19.089523	12.55858
**237.0301@11.548002**	6213.2295	40793.227	-11.1154
**910.5895@14.4609995**	179019.19	110802.97	94966.91
**298.1522@10.998998**	79170.98	27540.6	1403.218
**665.3774@14.526**	84806.27	66009.734	140005.6
**433.1104@11.046998**	65116.605	57927.45	10.16416
**903.4014@10.897002**	5850.067	5318.0244	-10.2136
**384.0724@7.732001**	-4247.4536	-4247.4536	10.42428
**506.2968@15.582**	3396.5596	4648.3154	-11.8669
**541.1014@9.895998**	61347.156	48750.895	1104.502
**903.4017@11.130998**	4285.8647	4070.7083	-11.4437
**711.2312@11.475001**	63430.223	8826.391	67.52856
**481.0769@11.511001**	34584.086	20135.436	1139.678
**664.4404@14.481002**	2914.5627	-12.84924	3384.009
**186.0454@12.231999**	-991.906	24.399052	11.22653
**341.193@11.543**	32256.059	21848.102	526.2276

Table S2. The MS/MS spectra of the tentatively identified components of *Lepidium peruvianum* extracts

No	Name of compound	MS/MS spectrum
1	Glucosinalbin	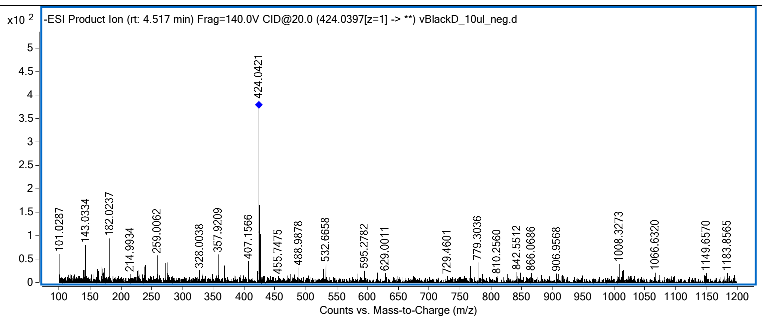
2	Glucotropaeolin	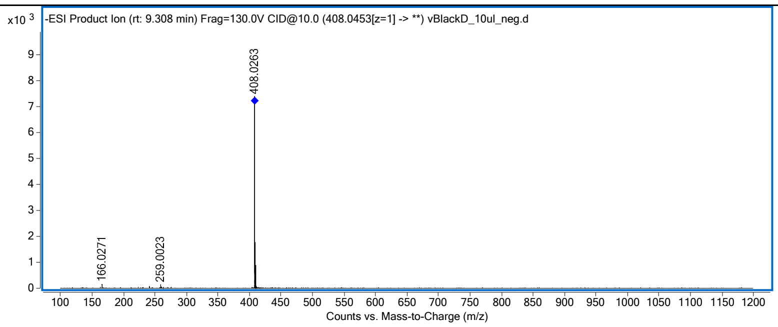
3	Glucolimnanthin	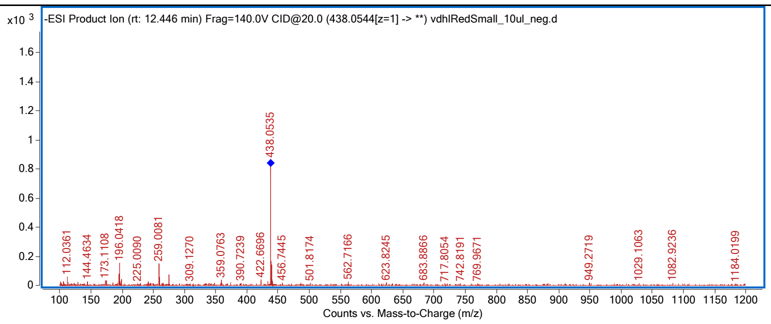
4	Pentenylglucosinolate	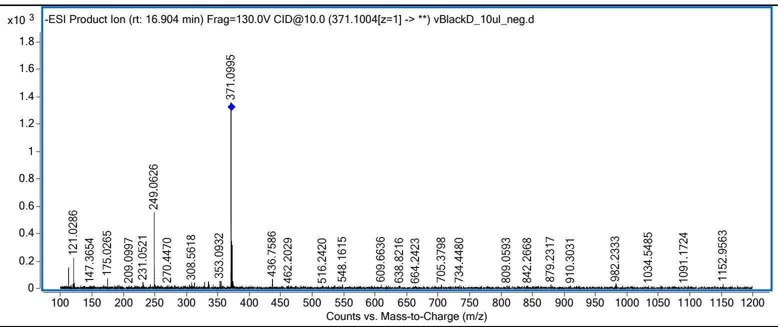
5	indolyl-hexyl-methyl-cyclohexane-glucosinolate	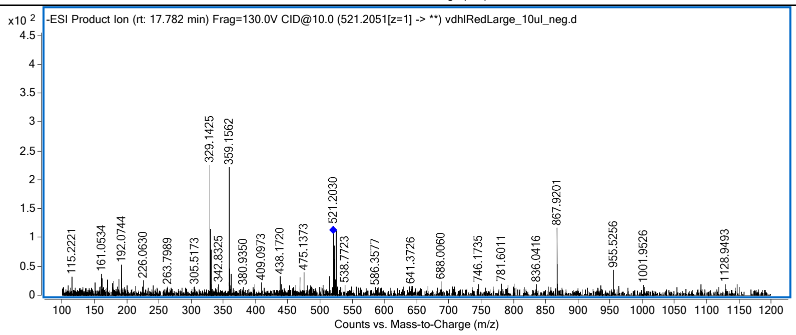

<table>
<tr><td>6</td><td>Lepidiline E</td><td>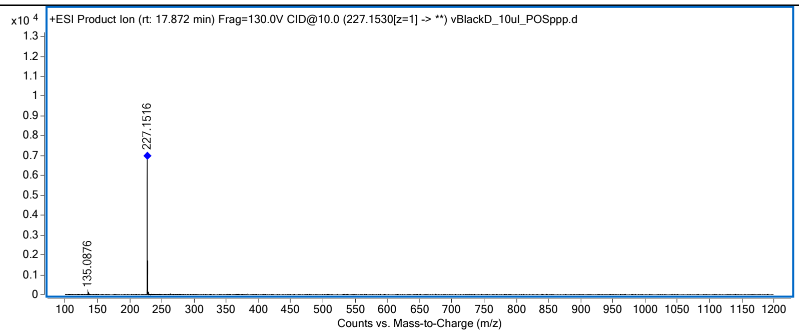
</td></tr>
<tr><td>7</td><td>Lepidiline A</td><td>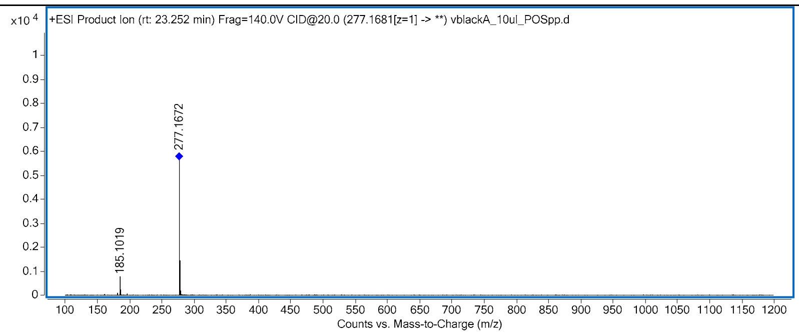
</td></tr>
<tr><td>8</td><td>Lepidiline C</td><td>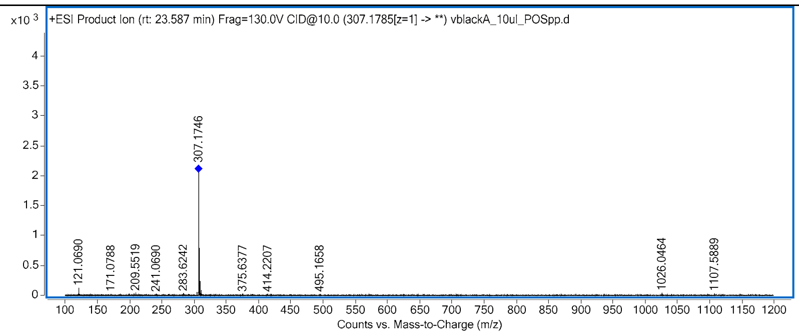
</td></tr>
<tr><td>9</td><td>Lepidiline D</td><td>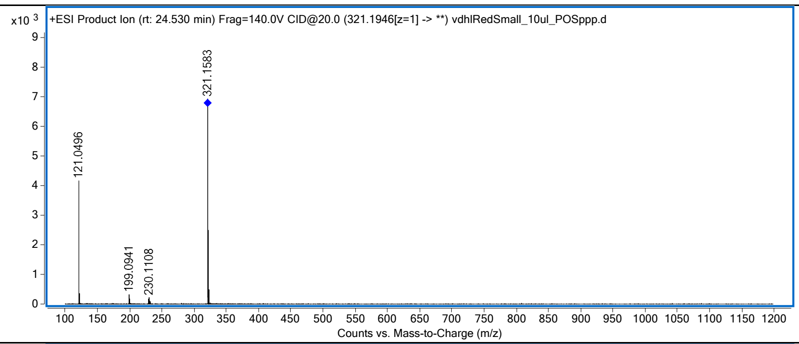
</td></tr>
<tr><td>10</td><td>Macamide B</td><td>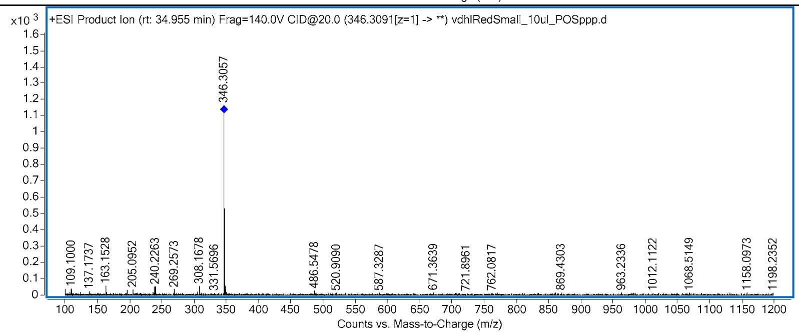
</td></tr>
</table>

11	Benzylo-oxo-octadecadienamide	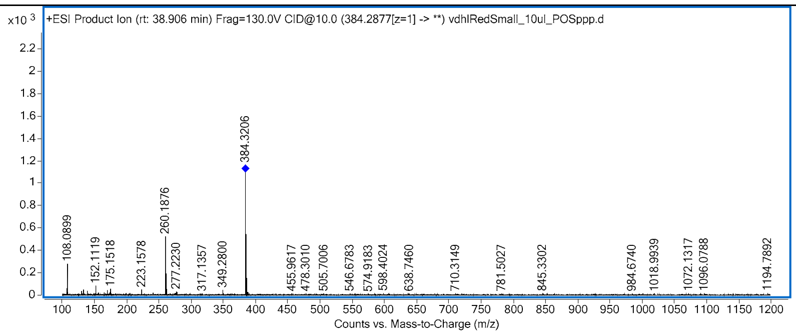

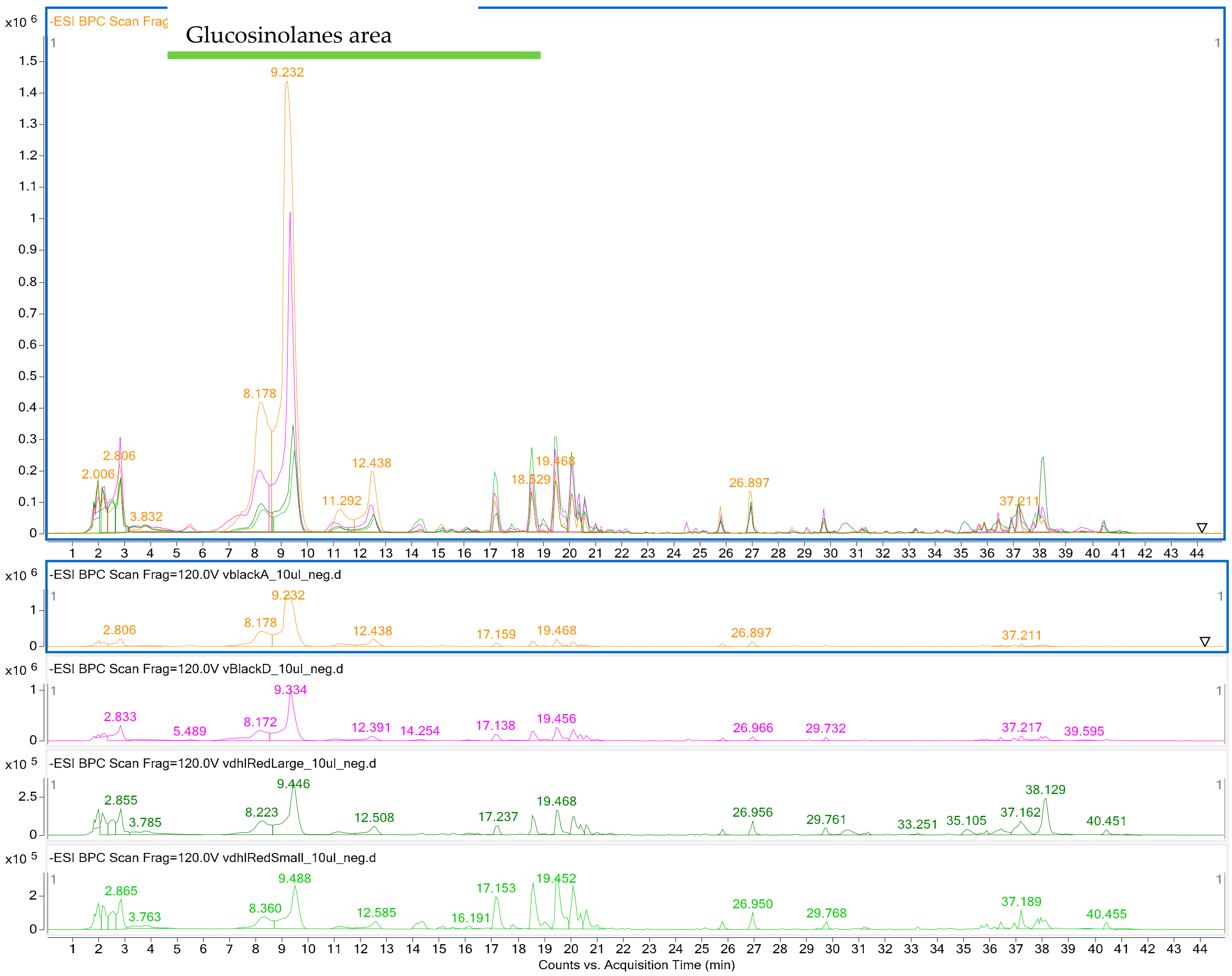

Figure S1. The total mass chromatograms recorded in the negative ionization mode for the tested maca samples.

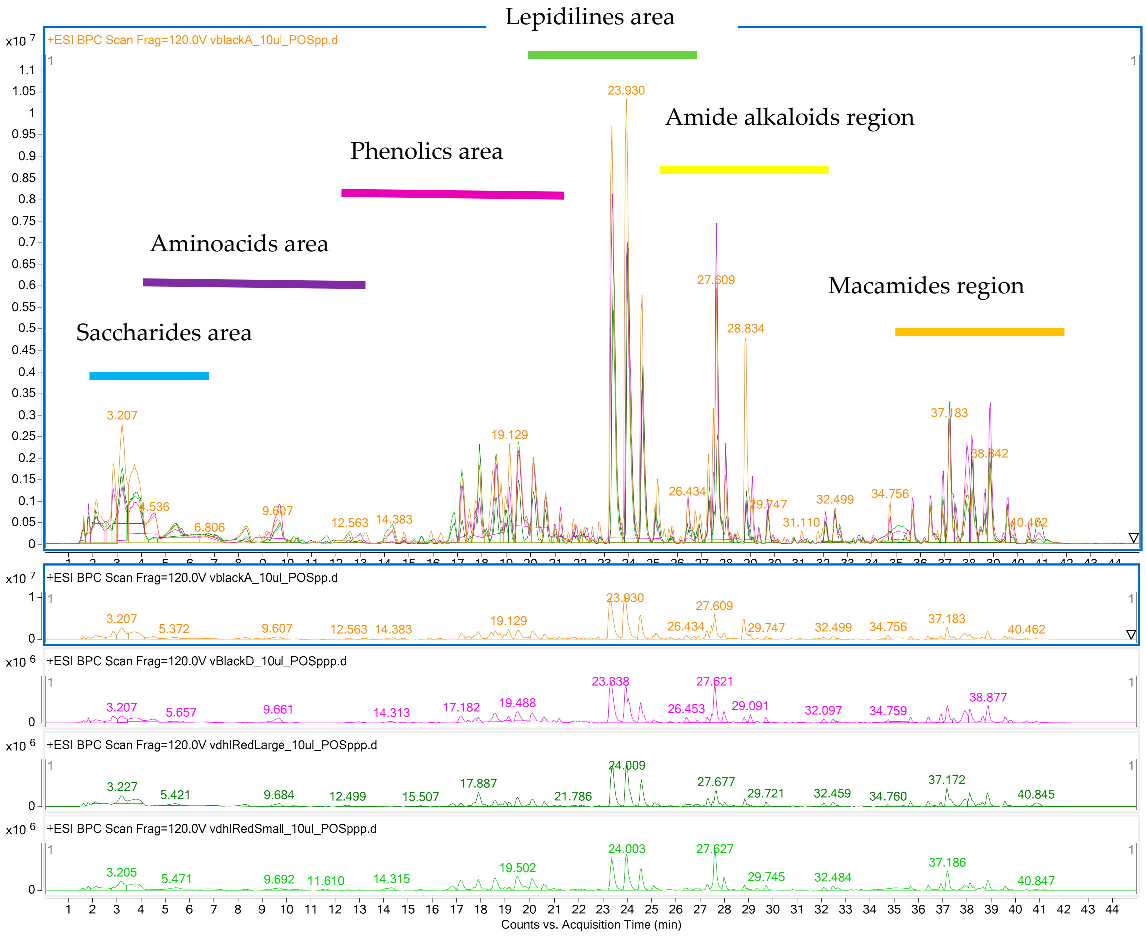

Figure S2. The total mass chromatograms recorded in the positive ionization mode for the tested maca samples.

Table S3. Raw data used for the volcano-type graphs, where the following cut-off points were used: FC +/- 10 and p value ≤ 0.001.

**Positive ionization mode**			
**Compound@retention time**	FC (BL_LARGE vs RE_SMALL)	FC (BL_SMALL vs RE_SMALL)	FC (RE_LARGE vs RE_SMALL)
**332.1582@12.779002**	-351582.53	-351583	-351583
**183.1081@8.895998**	944966.1	193905.6	292198.9
**332.1821@15.537998**	1952130.2	1344371	1780623
**394.1679@2.1199994**	1346078	789117.2	796640.4
**340.1479@11.869999**	202920.9	347487.3	164694.1
**332.188@13.166**	151019	361437.9	376447
**708.406@14.731998**	44953.305	2016271	391480
**637.1181@8.558999**	49217.72	335920.8	99725.36
**537.2063@9.862**	65774.266	122407.1	117900.9
**642.267@12.203002**	89673.41	37662.87	17886.46
**282.4973@11.561998**	118518.72	192161	31734.24
**250.1342@13.998**	165751.36	69259.63	49082.79

**Negative ionization mode**			
**Compound@retention time**	FC (BL_LARGE vs RE_SMALL)	FC (BL_SMALL vs RE_SMALL)	FC (RE_LARGE vs RE_SMALL)
**463.2547@10.4470005**	14152.336	9173.428	-10.7329
**876.1531@14.194**	-1057.2604	-1057.2604	26.2196
**846.1342@14.11**	-1108.4723	-1108.4723	33.48628
**333.0914@10.916**	-1174.285	19.089523	12.55858
**237.0301@11.548002**	6213.2295	40793.227	-11.1154
**910.5895@14.4609995**	179019.19	110802.97	94966.91
**298.1522@10.998998**	79170.98	27540.6	1403.218
**665.3774@14.526**	84806.27	66009.734	140005.6
**433.1104@11.046998**	65116.605	57927.45	10.16416
**903.4014@10.897002**	5850.067	5318.0244	-10.2136
**384.0724@7.732001**	-4247.4536	-4247.4536	10.42428
**506.2968@15.582**	3396.5596	4648.3154	-11.8669
**541.1014@9.895998**	61347.156	48750.895	1104.502
**903.4017@11.130998**	4285.8647	4070.7083	-11.4437
**711.2312@11.475001**	63430.223	8826.391	67.52856
**481.0769@11.511001**	34584.086	20135.436	1139.678
**664.4404@14.481002**	2914.5627	-12.84924	3384.009
**186.0454@12.231999**	-991.906	24.399052	11.22653
**341.193@11.543**	32256.059	21848.102	526.2276
